# Diagnostic Performance of Ankle-Brachial Pressure Index in Lower Extremity Arterial Disease

**DOI:** 10.1055/s-0041-1731444

**Published:** 2021-07-19

**Authors:** Mohammed Alagha, Thomas M. Aherne, Ahmed Hassanin, Adeel S. Zafar, Doireann P. Joyce, Waqas Mahmood, Muhammad Tubassam, Stewart R. Walsh

**Affiliations:** 1 Discipline of Vascular Surgery, National University of Ireland, Galway, Ireland

**Keywords:** ankle-brachial pressure index, peripheral arterial disease, duplex Doppler ultrasonography, CT angiography

## Abstract

**Introduction** Ankle-brachial pressure indices (ABIs) continue to form the basis of diagnostics for lower extremity arterial disease (LEAD). However, there remains a paucity of data to support its accuracy. This study aims to evaluate its diagnostic sensitivity and specificity using established arterial-imaging modalities as a benchmark.

**Methods** In this retrospective study, a regional, prospectively maintained, vascular laboratory database was interrogated to identify referred patients with arterial disease who underwent concomitant assessment with ABI and lower limb arterial duplex ultrasound (DUS). Duplex acted as the reference standard. Those who had peripheral computed tomography angiogram (CTA) within 3 months of initial assessment were included in a subgroup analysis to correlate ABI with CTA. The primary end point was the sensitivity and specificity of ABI compared with DUS as the reference standard.

**Results** Concomitant assessment was performed in 438 limbs (250 patients) over a 27-month period. The ABI was normal (0.9 to 1.4) in 196 limbs (44.9%) and abnormal in the remaining 241 limbs (55.1%). False-positive results occurred in 83 out of 241 limbs (34.4%), and false-negative results occurred in 54 limbs out of 196 (27.5%). True-positive results were 158 out of 241 limbs (65.6%), whereas true-negative results were 142 out of 196 limbs (72.4%). ABI using DUS as a benchmark identified a sensitivity for peripheral artery disease of 72.3% and a specificity of 69.3%. Concomitant CTA imaging was available in 200 limbs. The sensitivity and specificity of ABI correlated with CTA were 65.5 and 68.8%, respectively.

**Conclusion** ABIs have a moderate predictive value in the diagnosis of LEAD. Normal range outcomes cannot be taken to infer the absence of LEAD and, as such, further arterial imaging in the form of DUS or angiography should be strongly considered in those with suspected underlying disease requiring intervention. Further noninvasive tests such as exercise studies or pulse volume waveforms should be considered, if diagnostic uncertainty exists, in those requiring nonoperative intervention and risk factor control.

Lower extremity arterial disease (LEAD) is a manifestation of an underlying atherosclerotic process and may lead to a broad spectrum of clinical presentations ranging from asymptomatic occult disease to life-threatening limb ischemia. 1 The Intersociety Consensus Guidelines suggest that LEAD can be diagnosed noninvasively, using segmental blood pressure measurements, by obtaining an ankle-brachial pressure index (ABI). 1 2 Typically, it is assumed that, the ABI can reliably establish the diagnosis and severity of LEAD in the office setting, while offering a useful screening tool for those with asymptomatic disease. 3 Furthermore, it may monitor and predict functional decline in claudicants, 4 5 6 offer an objective measurement of procedural effects, 7 and provide insight into the etiology of lower limb ulceration thus expediting timely care decisions. 8 9 In this context of broad applicability, and presumed sensitivity and specificity, it is widely utilized particularly given the subjectivity associated with clinical examination. 10

However, despite the perceived benefits of ABI assessment, LEAD remains undiagnosed in over one-third of high-risk patients 11 potentially leading to significant cardiovascular-related morbidity. 12 These shortcomings, while multifactorial, are frequently a result of the subjective variability of both assessors and patients. 13 Characteristically an ABI of <0.9 is deemed diagnostic of underlying LEAD 14 15 with mild-to-moderate disease suggested by a measurement of between 0.4 and 0.9. An ABI less than 0.4 is suggestive of severe LEAD, 15 while a value greater than 1.4 is also considered abnormal, suggestive of noncompressible vessels.

Review data correlate the diagnostic uncertainty associated with ABI with Guirguis-Blake et al 16 reporting a low degree of sensitivity (7–34%) and an acceptable specificity of 96 to 100%, respectively, among 306 patients benchmarked against magnetic resonance angiography on behalf of the US Preventive Services Task Force. Indeed, recent Cochrane Review data examining the diagnosis of exertional leg pain further reflect this ambiguity suggesting that data to support the use of routine ABI is inadequate and recommend the generation of further cross-sectional data to establish its efficacy. 17 This diagnostic uncertainty, particularly in the comorbid arteriopathic cohort, has the potential for devastating consequences with missed opportunities for diagnoses precluding timely risk-factor and operative intervention.

In our unit, patients with suspected LEAD undergo concomitant ABI and arterial duplex ultrasound (DUS) to characterize underlying arterial disease. We aimed to evaluate the diagnostic performance of ABI in contemporary practice using both noninvasive DUS and the gold-standard peripheral computed tomography angiogram (CTA) as an objective benchmark of practice.

## Methods

In this retrospective study, a prospectively maintained database of patients attending a regional vascular laboratory in Galway University Hospital, Ireland, was interrogated to identify LEAD patients who underwent concomitant ABI and lower limb DUS over a 27-month period from January 2018 to April 2020. All patients undergoing dual assessment with ABI and DUS to investigate LEAD were included in the analysis. Dual assessment was indicated by a clinical suspicion of lower limb arterial disease in the presence of signs and symptoms of arterial disease and/or an abnormal lower limb examination or multiple cardiovascular risk factors. Patients with clinically palpable peripheral pulses, those undergoing a single modality of arterial investigation and those unable to undergo ABI (ulceration, amputation or pain), were excluded. Ethical approval for the conduct of research was granted by the Galway Clinical Research Ethics Committee (Reference: C.A. 2412)

The laboratory, operated by four fully accredited vascular technicians, serves a catchment area of 800,000 in the West and North-West of Ireland. Data regarding the patients' presentation, comorbidities, cardiovascular risk factors, and various cardiovascular complications (aneurysmal disease, ischemic heart disease, cerebrovascular disease) were obtained using patient medical notes. ABI was performed according to a standard dopplerometric protocol (using a handheld Doppler), by determining the higher systolic pressure in the two pedal arteries (dorsalis pedis and posterior tibial), and then dividing the figure attained by the brachial arterial systolic pressure. 18 19 In calculating the ABI, the higher of the two brachial systolic pressure measurements was used. Normal cutoff values for ABI were between 0.9 and 1.4 20 with values <0.9 and >1.4 deemed abnormal.

For the purposes of benchmarking arterial DUS acted as the reference standard, with a stenosis of greater than 50% in any of the iliac, femoropopliteal or infrapopliteal segments deemed to be diagnostic of LEAD. The degree of stenosis was defined on DUS by direct luminal size measurement in relation to the true lumen or by measurement of blood flow velocities at or above suspected sites of stenosis in each arterial segment. Any value above 125cm/sec or greater was considered to be indicative of a significant stenosis (50% or above). As DUS is operator-dependent, subgroup analyses were undertaken to assess the diagnostic performance of ABI compared with CTA in all those who underwent CTA within 3 months of the reference duplex scan.

All statistical analysis was performed using Statsdirect (Altrincham [StatsDirect Ltd, Merseyside, United Kingdom]). Descriptive statistics were used to summarize baseline characteristics of all participants. The diagnostic performance of ABI was evaluated using sensitivity, specificity, and receiver operating characteristic (ROC) curve analysis. Sensitivity refers to the proportion of patients who had significant stenosis as evident by DUS and CTA, if available, and had a positive ABI. Specificity was determined by the proportion of patients without evidence of significant stenosis, as evident on arterial imaging, and an associated negative ABI (normal) result. Sensitivity = true positive/(true positive + false negative), and specificity = true negative / (true negative + false positive).

## Results

**Fig. 1:**
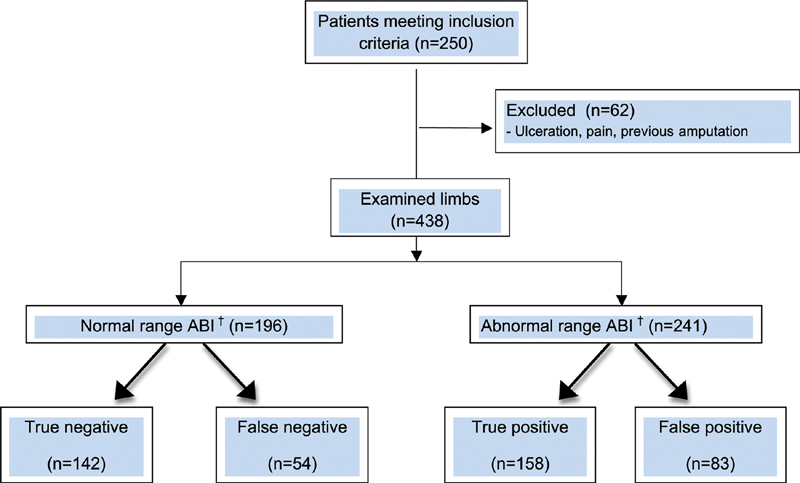
Flow diagram of included patients. †Abnormal refers to readings of <0.9 and >1.4; abnormal refers to readings between 0.9 and 1.4. ABI, ankle-brachial index.

ABI plus arterial duplex was performed in 438 limbs (250 patients) over a 27-month period. Sixty-two limbs were not assessed due to ulceration, pain, or previous amputation. Study flow is summarized in Fig. 1 .

The mean age of those assessed was 66.2 years with a predominance of male presentations (54%). In total 56.8% (142/250) of patients presented with critical limb ischemia (ongoing rest pain or tissue loss) with a prevalence of cardiovascular risk factors including diabetes (22%, 55/250), hypertension (59.6%, 149/250), and hyperlipidemia (46.4%, 116/250). Forty-six percent (115/250) patients had experienced at least one major cardiovascular event, while 48.4% (121/250) patients reported symptoms of life-limiting intermittent claudication. Eighty-two percent reported compliance with best medical therapy at the time of review.

**Fig. 2:**
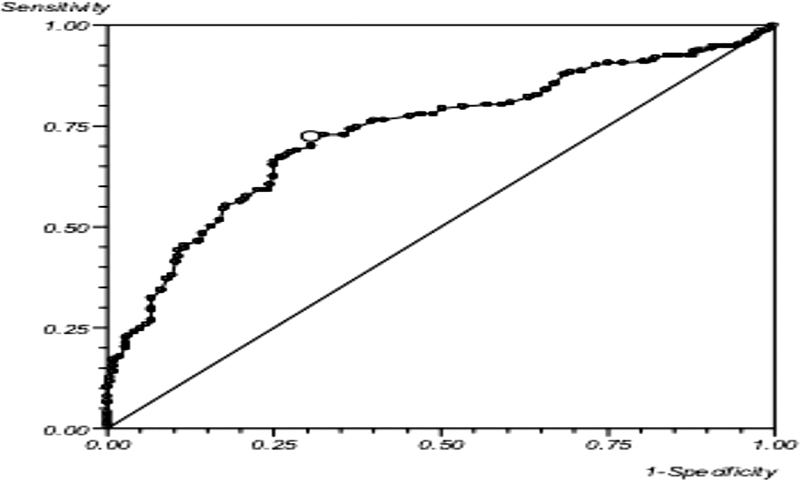
Receiver operating characteristic curve depicting the sensitivity and specificity of ankle brachial index using duplex ultrasound as a reference standard. x-axis depicts 1-specificity of the test; y-axis depicts sensitivity of the test.

The ABI was normal in 196 limbs (44.9%). Of these, 54 (27.5%) were noted to have a significant stenosis on DUS. Conversely 241 limbs (55.1%) were noted to have an abnormal ABI with 158 (65.6%) being identified as having significant stenosis on DUS ( Table 1 ). Data were incomplete for one limb and excluded. Calculation of diagnostic efficacy identified that ABI had a diagnostic sensitivity for LEAD of 72.3% and specificity was 69.3% when correlated with arterial DUS. The area under the ROC curve ( Fig. 2 ) for ABI was 0.73 (95% confidence interval [CI]: 0.63–0.83) indicating moderate predictive ability.

**Table 1:** ABI results with arterial duplex scan findings

Test (ABI)	Presence of significant stenosis (>50%)	Absence of significant stenosis (>50%)	Total
Positive (abnormal)	158	83	241
Negative (normal)	54	142	196
Total	212	225	437 a

Abbreviations: ABI, ankle-brachial index.

a One limb excluded due to incomplete data.

**Fig. 3:**
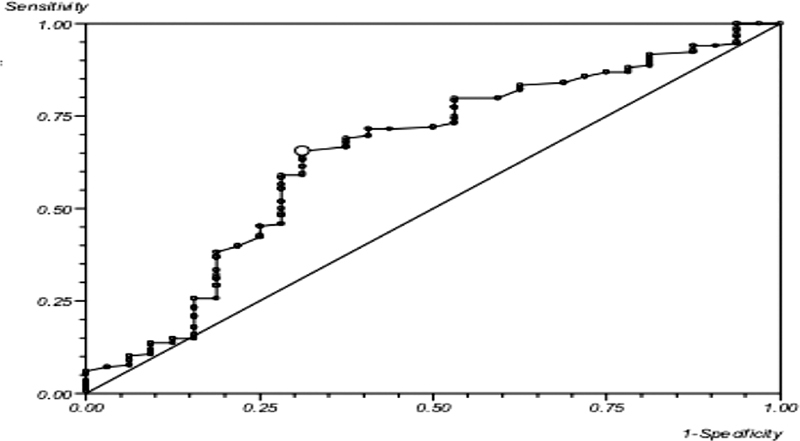
Receiver operating characteristic curve depicting the sensitivity and specificity of ankle brachial index using computed tomography angiography as a reference standard. x-axis depicts 1-specificity of the test; y-axis depicts sensitivity of the test.

Subgroup analysis of the 200 limbs undergoing adjunctive CTA provided objective data for correlation and a secondary point of reference. In this subset, ABI had an area under the ROC curve of 0.65 (95% CI: 0.49–0.80) ( Fig. 3 ) and a sensitivity and specificity of 65.5 and 68.8%, respectively, when compared with duplex.

**Fig. 4:**
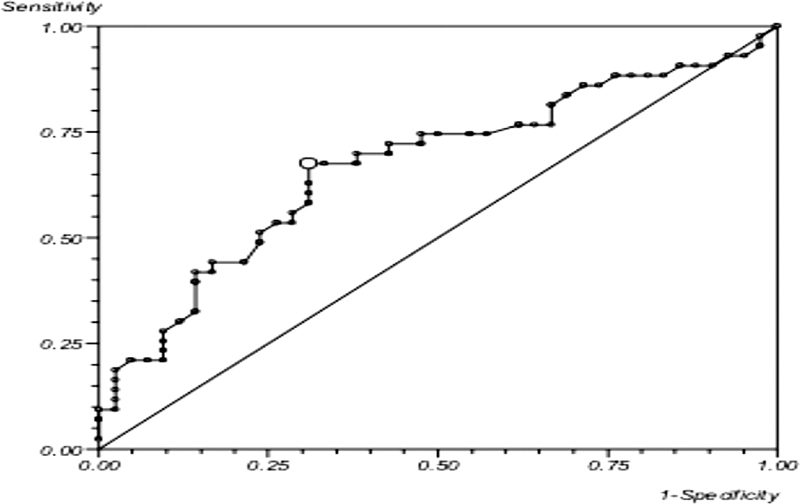
Receiver operating characteristic curve depicting the sensitivity and specificity of ankle brachial index, in the diabetic cohort, using duplex ultrasound as a reference standard. x-axis depicts 1-specificity of the test; y-axis depicts sensitivity of the test.

Further analysis of ABI in the diabetic cohort (86 limbs) revealed a sensitivity of 67.4% and a specificity of 69% using DUS as a reference standard with an area under the ROC curve of 0.67 (95% CI: 0.48–0.86) ( Fig. 4 ).

## Discussion

APIs remain a widely utilized diagnostic adjunct in the management of LEAD despite a scarcity of supportive data. This is likely related to its broad base of indications, simplicity of use, and it is relatively inexpensive. Additionally, an established safety profile and the limited resourcing its use requires ensures that it can be offered with ease in the community setting to those with both arterial and venous disease. These data examined the outcomes of 438 limbs, with clinical LEAD undergoing concomitant assessment with both ABI and arterial DUS. Notably, 27.5% of those deemed to have a normal ABI had evidence of significant arterial disease on DUS with sensitivity and specificity of ABI, using DUS as a benchmark, of 72.3 and 69.3%, respectively. Correlation of ABI performance with CTA outcomes revealed broadly similar outcomes with a sensitivity of 65.5% and a sensitivity of 68.8%.

Nonetheless definitive evidence to support routine ABI is limited. Recent European Society of Cardiology guidelines 21 suggest that ABI has a sensitivity of 75% and specificity of 86% for lower limb LEAD, based on established review data. 22 These data are, however, based on a small number ( *n* = 4) of heterogeneous studies exposing its outcomes to systematic bias. Additional Cochrane Review data from Crawford et al 17 screened over 17,000 citations in an attempt to identify cross-sectional studies comparing ABI to either diagnostic angiography or arterial DUS as reference standards. Only a single eligible study was identified assessing 85 participants (158 legs evaluated by untrained personnel) with a reported sensitivity and specificity of ABI of 95 and 56% using dopplerometric ABI in patients with leg pain. Similar diagnostic outcomes have also been identified using oscillometric ABI with meta-analysis data from 1,263 subjects suggesting a sensitivity of just 65% and a specificity of 96%. 23

These data correlate some of the diagnostic limitations associated with ABI identified in previous studies. 17 22 However, significant outcome heterogeneity exists among these reports with limited methodologically robust data to generate a definitive consensus. The current report is reflective of “real-world” practice in a high-volume tertiary unit. Fully trained laboratory personnel proficient in ABI and DUS provided validated assessment outcomes, while the inclusion of a CTA subgroup data offered a “gold-standard” to benchmark the actual diagnostic performance of ABI and remove the subjectivity of DUS assessment. All patients were undifferentiated at the time of assessment in the vascular laboratory thus limiting the effects of selection bias associated with specialist clinical assessment and referral. Despite these controlled conditions, the diagnostic sensitivity and specificity of ABI lower than could be reasonably expected, for an initial diagnostic test, when correlated with both DUS and CTA. Given the gravity of arterial disease, its associated sequelae, and the benefits of timely risk-factor modification, the associated false-negative rate exposes up to 38% of the evaluated population to potential undertreatment, while almost half of patients conceivably exposed to the unnecessary radiation and contrast dosages associated with the angiographic evaluation of false positives. Furthermore, the variations in sensitivities (72.3 vs. 69.3%) and specificities (65.5 vs. 68.8%) reported using both DUS and CTA as references in the CTA subgroup may be reflective of observer bias in those undergoing simultaneous ABI and DUS assessment.

Similar data from Bunte et al 24 again suggest that a significant proportion of patients (29%) with ischemic tissue loss may indeed have an ABI reading within normal range. These data suggest that toe pressure index may correlate more closely with infragenicular runoff than ABI and that a combination of both assessments may improve the noninvasive evaluation of significant ischemia; however, while this is implied by the authors, it is not statistically supported. Indeed, these limitations also potentially weaken the merits of ABI as a screening tool for asymptomatic LEAD with recent review data 25 identifying no population level data assessing the benefits or harms associated with ABI screening.

Further noninvasive investigative strategies, not examined in the current report, including postexercise arterial pressures, pulse volume waveforms, and ultrasound scoring systems potentially carry additional diagnostic yield. Indeed, Stein et al 26 identified that 31% of patients referred to a vascular surgeon with a normal resting ABI experienced a significant postexercise drop in pressure indicative of LEAD. Additionally, pulse volume waveforms, in diabetic populations, have been shown to carry higher diagnostic sensitivities (81.8%) when compared with other noninvasive tests including ABI measurements with plethysmography (20%) and Doppler (72.2%). 27

Interestingly, Santoro et al 28 29 report the use of a novel semiquantitative ultrasonographic scoring systems depicting atherosclerotic lesion characteristics and anatomy. These systems may be used to further inform clinicians with regard to unforeseen cardiovascular risk and thus guide risk factor modification. Moreover, the described scoring system has been shown to offer better cardiovascular risk quantification than ABI alone.

These described noninvasive tests offer additional diagnostic information at a lower morbidity profile than all forms of angiography and should potentially be considered in patient cohorts, where diagnostic uncertainty exists, who are likely to require nonoperative management of LEAD and to guide risk factor modification. Angiography should largely be reserved for those requiring invasive interventions.

This study is not without limitation. Its retrospective data capture exposes it to the innate bias of such reports with incomplete CTA data offering only partial benchmarking of the included ABI outcomes against the gold-standard. Similarly, the small sample size of this high-risk cohort limits the generalizability to those deemed to be at risk of LEAD. Importantly, insufficient data from the diabetic cohort limited the assessment of ABI in this important arterial group. Conversely these outcomes represent a large dataset in the context of ABI assessment as a diagnostic tool with sparse published data to support its use. The included benchmarking further strengthens the hypothesis that ABI, as both a diagnostic and screening tool, particularly in the high-risk LEAD patient group, should be supported by concomitant arterial DUS and/or angiographic imaging. Indeed, until its diagnostic performance is definitively determined and reliable diagnostic thresholds are established by powered, controlled studies, it may be more prudent to obtain vascular tree imaging to rule out LEAD in the absence of palpable pulses.

## Conclusion

ABIs have a moderate predictive value in diagnosis of LEAD. Normal range outcomes cannot be taken to infer the absence of LEAD and, as such, further arterial imaging in the form of DUS or angiography should be strongly considered in those with suspected underlying disease whom are likely to require operative intervention. Further noninvasive tests such as exercise studies or pulse volume waveforms should be considered, if diagnostic uncertainty exists, in those requiring nonoperative intervention and risk factor control.
